# Chemically Homogenous Compounds with Antagonistic Properties at All α_1_-Adrenoceptor Subtypes but not β_1_-Adrenoceptor Attenuate Adrenaline-Induced Arrhythmia in Rats

**DOI:** 10.3389/fphar.2016.00229

**Published:** 2016-08-03

**Authors:** Karolina Pytka, Klaudia Lustyk, Elżbieta Żmudzka, Magdalena Kotańska, Agata Siwek, Małgorzata Zygmunt, Agnieszka Dziedziczak, Joanna Śniecikowska, Adrian Olczyk, Adam Gałuszka, Jarosław Śmieja, Anna M. Waszkielewicz, Henryk Marona, Barbara Filipek, Jacek Sapa, Szczepan Mogilski

**Affiliations:** ^1^Department of Pharmacodynamics, Faculty of Pharmacy, Jagiellonian University Medical CollegeKrakow, Poland; ^2^Department of Pharmacobiology, Faculty of Pharmacy, Jagiellonian University Medical CollegeKrakow, Poland; ^3^Department of Medicinal Chemistry, Faculty of Pharmacy, Jagiellonian University Medical CollegeKrakow, Poland; ^4^Control and Robotics Group, Institute of Automatic Control, Faculty of Automatic Control, Electronics and Computer Science, Silesian University of TechnologyGliwice, Poland; ^5^Systems Engineering Group, Institute of Automatic Control, Faculty of Automatic Control, Electronics and Informatics, Silesian University of TechnologyGliwice, Poland; ^6^Department of Bioorganic Chemistry, Chair of Organic Chemistry, Faculty of Pharmacy, Jagiellonian University Medical CollegeKrakow, Poland

**Keywords:** arrhythmia, antiarrhythmic agents, α_1_-adrenolytics, 2-methoxyphenylpiperazine, α_1A_-adrenoceptor antagonist, α_1B_-adrenoceptor antagonist, α_1D_-adrenoceptor antagonist, hypotensive

## Abstract

Studies proved that among all α_1_-adrenoceptors, cardiac myocytes functionally express only α_1A_- and α_1B_-subtype. Scientists indicated that α_1A_-subtype blockade might be beneficial in restoring normal heart rhythm. Therefore, we aimed to determine the role of α_1_-adrenoceptors subtypes (i.e., α_1A_ and α_1B_) in antiarrhythmic effect of six structurally similar derivatives of 2-methoxyphenylpiperazine. We compared the activity of studied compounds with carvedilol, which is β_1_- and α_1_-adrenoceptors blocker with antioxidant properties. To evaluate the affinity for adrenergic receptors, we used radioligand methods. We investigated selectivity at α_1_-adrenoceptors subtypes using functional bioassays. We tested antiarrhythmic activity in adrenaline-induced (20 μg/kg i.v.), calcium chloride-induced (140 and 25 mg/kg i.v.) and barium chloride-induced (32 and 10 mg/kg i.v.) arrhythmia models in rats. We also evaluated the influence of studied compounds on blood pressure in rats, as well as lipid peroxidation. All studied compounds showed high affinity toward α_1_-adrenoceptors but no affinity for β_1_ receptors. Biofunctional studies revealed that the tested compounds blocked α_1A_-stronger than α_1B_-adrenoceptors, but except for HBK-19 they antagonized α_1A_-adrenoceptor weaker than α_1D_-subtype. HBK-19 showed the greatest difference in pA_2_ values—it blocked α_1A_-adrenoceptors around seven-fold stronger than α_1B_ subtype. All compounds showed prophylactic antiarrhythmic properties in adrenaline-induced arrhythmia, but only the activity of HBK-16, HBK-17, HBK-18, and HBK-19 (ED_50_ = 0.18–0.21) was comparable to that of carvedilol (ED_50_ = 0.36). All compounds reduced mortality in adrenaline-induced arrhythmia. HBK-16, HBK-17, HBK-18, and HBK-19 showed therapeutic antiarrhythmic properties in adrenaline-induced arrhythmia. None of the compounds showed activity in calcium chloride- or barium chloride-induced arrhythmias. HBK-16, HBK-17, HBK-18, and HBK-19 decreased heart rhythm at ED_84_. All compounds significantly lowered blood pressure in normotensive rats. HBK-18 showed the strongest hypotensive properties (the lowest active dose: 0.01 mg/kg). HBK-19 was the only compound in the group, which did not show hypotensive effect at antiarrhythmic doses. HBK-16, HBK-17, HBK-18, HBK-19 showed weak antioxidant properties. Our results indicate that the studied 2-methoxyphenylpiperazine derivatives that possessed stronger α_1A_-adrenolytic properties (i.e., HBK-16, HBK-17, HBK-18, and HBK-19) were the most active compounds in adrenaline-induced arrhythmia. Thus, we suggest that the potent blockade of α_1A_-receptor subtype is essential to attenuate adrenaline-induced arrhythmia.

## Introduction

Arrhythmias are the most common causes of sudden cardiac death (Deo and Albert, 2012). Despite numerous antiarrhythmic drugs, pharmacotherapy is still ineffective in majority of patients. Moreover, all antiarrhythmic agents acting via different ion channels possess life-threatening proarrhythmic potential. Thus, scientists are still looking for effective and safe compounds, which will protect against arrhythmia and/or restore normal heart rhythm.

Antiarrhytmic activity of pharmacological agents resulting from their receptor-based mechanisms might be equally efficient and much safer than that observed for classical antiarrhythmic drugs. According to many studies, α_1_-adrenolytics may have potential in the treatment of arrhythmias. Scientists agree that the blockade of α_1_-, and particularly α_1A_-adrenoceptor may be beneficial in restoring normal heart rhythm (reviewed in Hieble, 2000 and Shannon and Chaudhry, 2006). The α_1_-adrenoceptor stimulation results in inositol trisphosphate (IP_3_) production, and subsequent Ca^2+^ release from the sarcoplasmatic reticulum (SR; Escobar et al., 2012). Although the regulation of Ca^2+^ level in cardiomyocytes mainly depends on ryanodine receptors and SR Ca^2+^ pump, the increased basal level of Ca^2+^ induced by IP_3_ may also alter the electrical excitability of cardiomyocytes, thus contributing to the development of arrhythmia e.g., atrial (Zima and Blatter, 2004) or ventricular fibrillation (Proven et al., 2006). Thereby, the blockade of α_1_-adrenoceptors may lead to the stabilization of Ca^2+^ level producing antiarrhythmic effect in arrhythmias induced by catecholamines e.g., catecholaminergic polymorphic ventricular tachycardia. The above hypothesis was supported by Suita et al. (2015), who demonstrated that prazosin not only shortened norepinephrine-induced elongation of atrial fibrillation in mice, but also attenuated norepinephrine-induced SR Ca^2+^ leak and spontaneous SR Ca^2+^ release in cultured atrium cardiomyocytes. This proves that α_1_-adrenoceptors may have role in preventing cardiac arrhythmias. Numerous animal studies confirmed this theory, showing antiarrhythmic properties of α_1_-adrenolytics (Sapa et al., 2011; Kubacka et al., 2013a; Rapacz et al., 2014, 2015b).

Since in our earlier experiments 2-methoxyphenylpiperazine derivatives showed high affinity toward α_1_-adrenoceptors (Pytka et al., 2015), in this study we aimed to determine the role of α_1_-adrenoceptors subtypes (i.e., α_1A_, α_1B_) in antiarrhythmic effect of six structurally similar derivatives of 2-methoxyphenylpiperazine. We compared the activity of studied compounds with carvedilol, which is β_1_- and α_1_-adrenoceptors blocker with antioxidant properties.

## Materials and methods

### Animals

The experiments were carried out using male normotensive Wistar rats [Krf: (WI) WU], weighing 200–250 g. Animals were kept in plastic cages (three rats per cage) at constant room temperature of 22 ± 2°C, with 12:12 h light/dark cycle. Rats had free access to food (standard pellet diet) and water. Each experimental and control groups consisted of four to six animals. The animals were killed by cervical dislocation immediately after the experiment. All injections were given in a volume of 1 ml/kg. All experimental procedures were approved by the Local Ethics Committee for Experiments on Animals of the Jagiellonian University in Krakow, Poland (approval numbers 110/2014 and 246/2015) and cared for in accordance with the Guide to the Care and Use of Experimental Animals.

### Drugs

**Figure 1:**
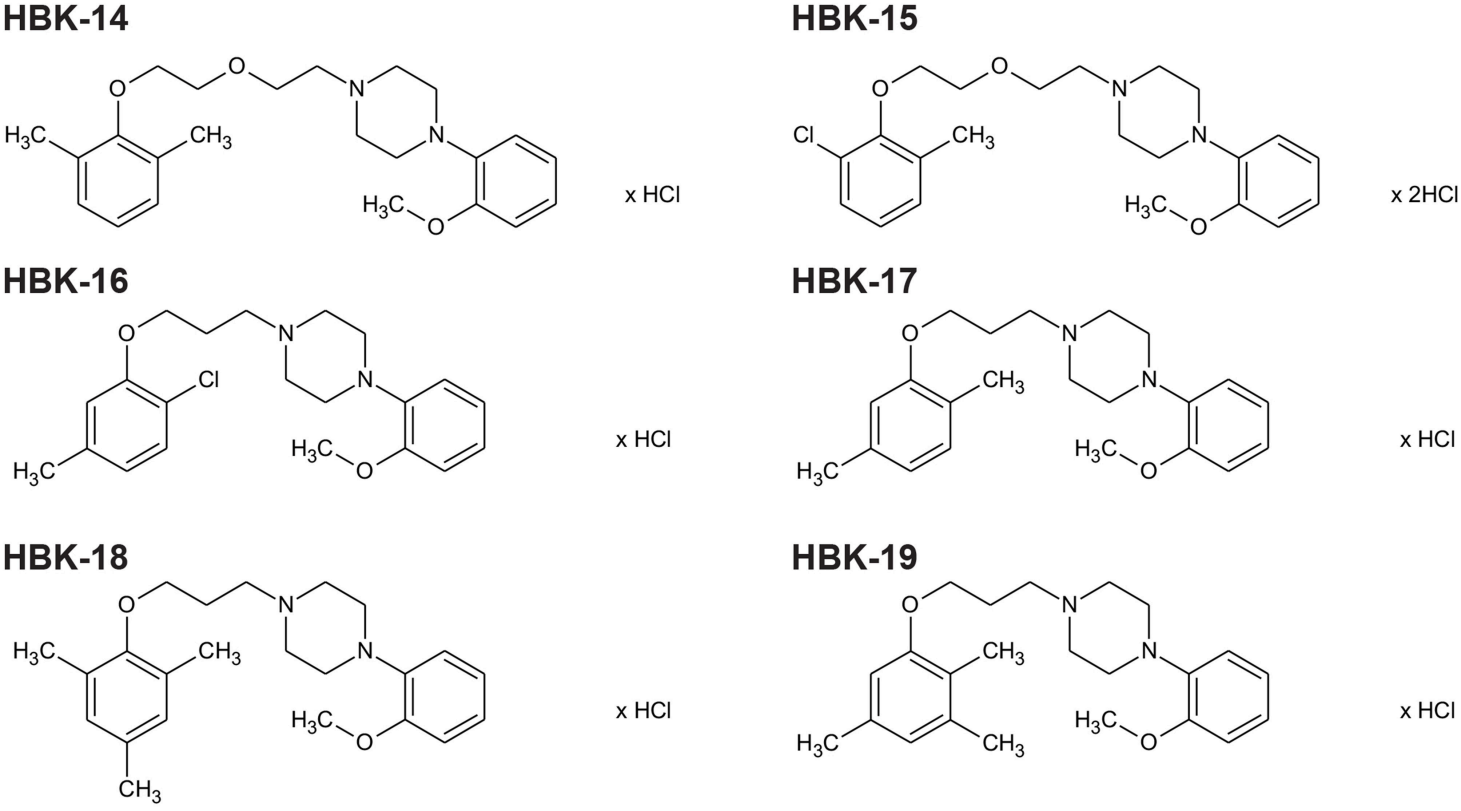
**The chemical structures of tested 2-methoxyphenylpiperazine derivatives**. HBK-14, 1-[(2,6-dimethylphenoxy)ethoxyethyl]-4-(2-methoxyphenyl)piperazine hydrochloride; HBK-15, 1-[(2-chloro-6-methylphenoxy)ethoxyethyl]-4-(2-meth oxyphenyl)piperazine hydrochloride; HBK-16, 1*N*-[3-(2-chloro-5-methylph enoxy)propyl]-4*N*-(2-methoxyphenyl)piperazine hydrochloride; HBK-17, 1*N*-[3-(2,5-dime thylphenoxy)propyl]-4*N*-(2-methoxyphenyl)piperazine hydrochloride; HBK-18, 1*N*-[3-(2,4,6-trimethylphenoxy)propyl]-4*N*-(2-methoxyphenyl)piperazine hydrochloride; HBK-19, 1*N*-[3-(2,3,5-trimethylphenoxy)propyl]-4*N*-(2-methoxyphenyl)piperazine hydrochloride.

Six studied compounds (Figure 1): 1-[(2,6-dimethylphenoxy)ethoxyethyl]-4-(2-methoxyphenyl)piperazine hydrochloride (HBK14), 1-[(2-chloro-6-methylphenoxy)ethoxyethyl]-4-(2-methoxyphenyl)piperazine hydrochloride (HBK15), 1*N*-[3-(2-chloro-5-methylphenoxy)propyl]-4*N*-(2-methoxyphenyl)piperazine hydrochloride (HBK16), 1*N*-[3-(2,5-dimethylphenoxy)propyl]-4*N*-(2-methoxyphenyl)piperazine hydrochloride (HBK17), and 1*N*-[3-(2,4,6-trimethylphenoxy)propyl]-4*N*-(2-methoxyphenyl)piperazine hydrochloride (HBK18), 1*N*-[3-(2,3,5-trimethylphenoxy)propyl]-4*N*-(2-methoxyphenyl)piperazine hydrochloride (HBK-19) were synthesized in the Department of Bioorganic Chemistry, Chair of Organic Chemistry, Faculty of Pharmacy, Jagiellonian University (Waszkielewicz et al., 2015). The investigated compounds were dissolved in saline and administered intraperitoneally (i.p.) or intravenously (i.v.). Thiopental (Rotexmedica, Germany) was dissolved in saline and administered i.p. Chloroethylclonidine (Sigma, Germany), noradrenaline (Sigma, Germany), johimbine (Tocris, United Kingdom), propranolol (Fluka, USA), phentolamine (Sigma, Germany) were dissolved in saline or dimethyl sulfoxide (DMSO, Sigma, Germany) and used in radioligand or biofunctional studies. Adrenaline (Polfa S.A., Warsaw), carvedilol (Sigma, Germany), methoxamine (Sigma, China), calcium chloride (Fluka, Germany), and barium chloride (Sigma, Germany) were dissolved in saline and administered i.v. Heparin (Polfa S.A., Warsaw) was used as anticoagulant during experiments. The control groups received 0.9% NaCl solution. Other chemicals used were obtained from POCh (Polish Chemical Reagents, Poland).

### Radioligand binding assay

The α_1_- and β_1_-adrenoceptor radioligand binding assay was performed on rat cerebral cortex. [^3^H]-prazosin (19.5 Ci/mmol, α_1_-adrenoceptor) and [^3^H]-CGP-12177 (48 Ci/mmol, β_1_-adrenergic receptor) were used. The brains were homogenized in 20 volumes of an ice-cold 50 mM Tris-HCl buffer (pH 7.6) and were centrifuged at 20,000 × g for 20 min (0−4°C). The cell pellet was resuspended in the Tris–HCl buffer and centrifuged again. Radioligand binding assays were performed in plates (MultiScreen/Millipore). The final incubation mixture (final volume 300 μl) consisted of 240 μl of the membrane suspension, 30 μl of [^3^H]-prazosin (0.2 nM), [^3^H]-CGP-12177 (0.2 nM) solution and 30 μl of the buffer containing seven to eight concentrations (10^−11^−10^−4^ M) of the tested compounds. In order to measure the unspecific binding, 10 μM phentolamine (for [^3^H]-prazosin) and 1 μM of propranolol (for [^3^H]-CGP-12177) were applied. The incubation was terminated by rapid filtration through glass fiber filters (Whatman GF/C) using a vacuum manifold (Millipore). The filters were then washed twice with the assay buffer and placed in scintillation vials with a liquid scintillation cocktail. Radioactivity was measured in a WALLAC 1409 DSA liquid scintillation counter (Perkin Elmer, USA). All the assays were made in duplicate and the inhibitory constants (K_i_) were estimated.

### Influence on α_1A_-adrenoceptors: rat tail artery

Rats were anesthetized with thiopental (75 mg/kg i.p.) and the middle part of the ventral caudal artery was removed, cleaned of surrounded tissues and uncovered of endothelium by gentle rubbing in the Krebs-Henseleit solution. The isolated artery, cut into ~4 mm long rings, was then horizontally put up between two stainless steel hooks (diameter 0.15 mm). One hook was fastened to the bottom of the chamber and the other to an isometric FDT10-A force displacement transducer (DMT Model 750TOBS, Denmark), linked with Powerlab 4/26 analyzer (ADInstruments), and processed by LabChart 7 software. The isolated rings were incubated in the Krebs-Henseleit solution (20 ml) at the temperature 37°C and pH 7.4 with constant oxygenation (O_2_/CO_2_, 19:1). The initial optimal tissues tension was set at 0.75 g. Chloroethylclonidine (3 μM) as the preferential α_1B_-adrenoreceptor alkylating agent was used during incubation and after 30 min it was completely washed off. Through 100 min of equilibration tissues were stimulated with noradrenaline (1 μM) four times with washing out until the contractile response become constant. Two cumulative concentration-response curves to noradrenaline, at an interval of 60 min, were established on each arterial ring both in the presence and absence of antagonist. The incubation with antagonists went on for 30 min. The experiments were carried out in the constant presence of yohimbine (0.1 μM) and propranolol (1 μM) to block α_2_- and β-adrenoceptors, respectively in order to minimize the involvement of these adrenoceptors in the response to noradrenaline.

### Influence on α_1B_-adrenoceptors: mouse spleen

The influence on α_1B_-adrenoceptors was evaluated using the isolated mouse spleen. The spleen was removed from male mice right after killing the anesthetized animal by cervical dislocation. The isolated tissue was incubated in 20 ml cup filled with the Krebs-Henseleit solution at the temperature 37°C and pH 7.4 with constant oxygenation (O_2_/CO_2_, 19:1). The initial optimal tissues tension was set at 1.0 g. Through the 100 min of equilibration tissues were stimulated with noradrenaline (0.1−10.0 μM) three times with washing out until the contractile response become constant. Two cumulative concentration-response curves to noradrenaline, at an interval of 60 min, were established on each tissue both in the presence and absence of antagonist. The incubation with antagonists went on for 30 min. The experiments were carried out in the constant presence of propranolol (1 μM) to block β-adrenoceptors, and minimize the involvement of these adrenoceptors in the response to noradrenaline.

### Influence on α_1D_-adrenoceptors: rat aorta

Finally, the influence on α_1D_-adrenoreceptors was investigated using the isolated rat aorta. Rats anesthetized with thiopental and killed by cervical dislocation had aorta removed, denuded of endothelium and incubated in the Krebs-Henseleit solution at the temperature 37°C and pH 7.4 with constant oxygenation (O_2_/CO_2_, 19:1). The aorta rings were maintained at optimal tension of 2.0 g. During 3 h of equilibration tissues were stimulated with noradrenaline three times (0.3 μM). Two cumulative concentration-response curves to noradrenaline, at an interval of 60 min, were established on each tissue both in the presence and absence of antagonist. The incubation with antagonists went on for 30 min. The experiments were carried out in the constant presence of yohimbine (0.1 μM) and propranolol (1 μM) to block α_2_- and β-adrenoceptors, respectively.

### Prophylactic antiarrhythmic activity in adrenaline-, barium chloride-, and calcium chloride-induced arrhythmias

The procedures were performed according to the method described by Szekeres and Papp (1975). The heart rate disturbances were evoked by the intravenous administration of adrenaline (20 μg/kg), barium chloride (32 and 10 mg/kg) or calcium chloride (140 and 25 mg/kg) solution into the caudal vein in anesthetized rats (thiopental, 75 mg/kg). The studied compounds were administered i.p. 45 min before the injection of adrenaline, calcium chloride, or barium chloride. The electrocardiogram (ECG) was recorded during the first 2 min as well as in the 5th, 10th, and 15th min after the arrhythmogen injection. The criterion of antiarrhythmic activity was the lack of extrasystoles and inhibition of cardiac arrhythmia in comparison with the control group in adrenaline-induced arrhythmia or the progressive disappearance of the arrhythmia and reinstatement of the sinus rhythm in barium chloride- and calcium chloride-induced arrhythmias. The ED_50_ (a dose producing a 50% inhibition of ventricular contractions) with 95% confidence limits was determined by computer log-probit analysis according to Litchfield and Wilcoxon (1949) and Szekeres and Papp (1975). The compounds were administered at the dose 10 mg/kg. We gradually decreased the dose by half until the disappearance of antiarrhythmic activity.

### Therapeutic antiarrhythmic activity in adrenaline-induced arrhythmia

The experiment was performed according to the method described by Szekeres and Papp (1968). The arrhythmia was evoked by the intravenous administration of adrenaline (20 μg/kg) into the caudal vein in anesthetized rats (thiopental, 75 mg/kg). The tested compounds were injected i.v. at the peak of arrhythmia directly after adrenaline injection, at the ED_84_ (a dose producing a 84% inhibition of premature ventricular contractions established in prophylactic adrenaline-induced arrhythmia). The range of doses was 0.325−0.504 mg/kg. The ECG was recorded constantly for 5 min as well as in the 10th and 15th min of the experiment. The criterion of antiarrhythmic activity was the reduction of premature ventricular contractions in comparison with the control group (Sapa et al., 2011).

### The effect on normal electrocardiogram in rats

The experiment was carried out to exclude the influence of tested compounds on normal ECG. The ECG was recorded (ASPEL ASCARD B5 apparatus, standard lead II and paper speed of 50 mm/s) prior and also 5, 10, 15, 20, 30, 40, 50, and 60 min after the i.p. administration of tested compounds. The influence on QRS complex, PQ interval, heart rate (RR), and QTc interval was determined. The QTc was calculated according to the Bazzett's formula: QTc = QT/√RR (De Clerck et al., 2002). The compounds were administered at the ED_84_ (a dose producing a 84% inhibition of premature ventricular contractions established in prophylactic adrenaline-induced arrhythmia).

### Influence on blood pressure in normotensive rats

Normotensive rats were anesthetized with thiopental (75 mg/kg ip). The right carotid artery was cannulated with a polyethylene tube filled with heparin solution to allow pressure measurements, using a Datamax apparatus (Columbus Instruments, USA; Kubacka et al., 2013b). The tested compounds were administered i.p. after 15 min of stabilization period. The compounds were administered at the dose 10 mg/kg. We gradually decreased the dose by half until the disappearance of antiarrhythmic activity.

### Influence on blood vasopressor response in rats

To verify if the hypotensive activity was a result of α-adrenolytic properties, we studied the influence of tested compounds on the pressor response to methoxamine (150 μg/kg) The experiment was carried out for all active compounds, which were administered (i.v.) to the caudal vein at the lowest hypotensive dose (Kubacka et al., 2013b). Pressor response to methoxamine injected i.v. was measured before (control) and 5 min after the tested compounds. We administered the tested compounds at the lowest possible doses, not to lose selectivity for α_1_-adrenoceptors.

### Antioxidant effect—lipid peroxidation in rat brain homogenate

This experiment was performed according to the method described by Yue et al. (1992). The rat brain homogenate containing 10 mg tissue/ml was prepared in 0.9% saline. The rates of membrane lipid peroxidation were measured by the formation of thiobarbituric acid reactive substances (TBARS). Rat brain homogenates (1 ml) were incubated at 37°C for 5 min with 10 μl of a tested compound or vehicle. Lipid peroxidation was initiated by the addition of 50 μl of 0.5 mM FeCl_2_ and 50 μl of 2.0 mM ascorbic acid. After 30 min of incubation, the reaction was stopped by adding 0.1 ml of 0.2% butylated hydroxytoluene (BHT). Thiobarbituric acid reagent was then added and the mixture was heated for 15 min in a boiling water bath. Carvedilol was used as reference compound. The compounds were tested at a concentration of 10^−3^ M. The TBARs were measured at 532 nm.

### Data analysis

In radioligand binding studies, the obtained data were fitted to a one-site curve-fitting equation with Prism 6.0 (GraphPad Software), and inhibition constants (K_i_) values were estimated from the Cheng−Prusoff equation (Cheng and Prusoff, 1973):

$$\begin{array}{l} K_{i}=\frac{IC_{50}}{1+\frac{L_{O}}{K_{D}}} \end{array}$$

*L*_O_–labeled ligand concentration, *K*_D_–dissociation constant of labeled ligand

In functional bioassays the concentration–response curves were analyzed using GraphPad Prism 6.0 software (GraphPad Software Inc., San Diego, CA, USA) as previously described by Kubacka et al. (2013b). Data are means ± S.E.M. of at least 4 separate experiments. To establish Hill slopes for the agonist concentration–response curves and calculate EC_50_ values, curves were fitted to all the data by non-linear regression. The EC_50_ value in the presence and absence of antagonists was used to ascertain the concentration ratio (CR). Schild analysis was performed. If the slope was not significantly different from unity, the relative antagonistic potencies (pA_2_: −log of the concentration of an antagonist that doubles the concentration of the agonist needed to elicit the original submaximal response obtained in the absence of antagonist) were determined by plotting the log (CR-1) against the −log of antagonist concentration (Arunlakshana and Schild, 1959).

In case of *in vivo* experiments the results are presented as the means ± S.E.M. Statistically significant differences between groups were calculated using one-way analysis of variance (ANOVA) with repeated measurements followed by Dunnett's or Bonferroni's test *post-hoc* or Student's *t*-test. The criterion for significance was set at *p* < 0.05.

Antioxidant activity was expressed as the percentage reduction of the sample absorbance during the reaction at wavelength 532 nm.

The log-probit method described by Litchfield and Wilcoxon (1949) was used to determine median effective doses (ED_50_—doses producing 50% inhibition of premature ventricular contractions) and doses producing 84% of the maximal effect (ED_84_) for compounds in arrhythmia models.

## Results

### Affinity for adrenoceptors

All studied compounds possessed high affinity for α_1_-adrenoceptors but none of them bound to β_1_-adrenoceptors (Table 1).

**Table 1:** **The affinity of tested compounds for α_**1**_- and β_**1**_-adrenoceptors**.

**Compound**	**Adrenergic receptors K**_i_ **(nM)**	
	**α_1_**	**β_1_**
HBK-14	22.8^a^	n.a^a^
HBK-15	13.1^a^	n.a^a^
HBK-16	5.0	n.a.
HBK-17	12.9	n.a.
HBK-18	5.2	n.a.
HBK-19	22.7	n.a.
Phentolamine	18.3	−
Propranolol	−	7.1
Carvedilol	2.2^b^	0.8^b^

*Inhibition constants (K_i_) were calculated according to the equation of Cheng and Prusoff (1973). The compounds were tested in three separate experiments in duplicates. n.a., compound did not bind to the receptor at the concentration 10^−5^ M*.

a *Pytka et al. (2015)*.

b *Pönicke et al. (2002)*.

### Functional affinity for α_1A_-, α_1B_-, and α_1D_-adrenoceptors

The tested compounds antagonized noradrenaline evoked contraction in isolated rat aorta and tail artery, as well as mouse spleen, and concentration-dependently, shifted the noradrenaline response to the right, without affecting the maximum response. The obtained pA_2_ values with Schild slopes not significantly different from unity indicated a competitive antagonism at α_1A_-, α_1B_-, and α_1D_-adrenoceptors (Tables 2A,B).

**Table 2A:** **The functional affinities of tested and reference compounds for α_**1**_-adrenergic receptor subtypes**.

**Compound**	**Isolated tissues, α_1_-adrenoceptor subtypes, pA_2_ ± S.E.M. (slope ± S.E.M.)**		
	**Rat tail artery**	**Mouse spleen**	**Rat aorta**
	**α_1A_**	**α_1B_**	**α_1D_**
HBK-14	7.99 ± 0.09 (0.97 ± 0.15)	7.70 ± 0.08 (1.03 ± 0.03)	8.86 ± 0.06 (1.09 ± 0.10)
HBK-15	7.71 ± 0.07 (1.01 ± 0.18)	7.67 ± 0.09 (0.96 ± 0.01)	8.90 ± 0.09 (1.09 ± 0.09)
HBK-16	8.75 ± 0.03 (1.06 ± 0.08)	8.21 ± 0.07 (1.03 ± 0.12)	**9.14** ± **0.03 (1.11** ± **0.12)**
HBK-17	8.15 ± 0.08 (0.91 ± 0.05)	7.94 ± 0.06 (0.91 ± 0.03)	8.83 ± 0.09 (1.01 ± 0.07)
HBK-18	8.49 ± 0.09 (1.10 ± 0.06)	**8.35** ± **0.05 (1.03** ± **0.18)**	8.92 ± 0.08 (1.01 ± 0.05)
HBK-19	**8.91** ± **0.09 (0.96** ± **0.05)**	8.07 ± 0.07 (1.06 ± 0.05)	8.31 ± 0.08 (1.03 ± 0.09)
Prazosin	8.93 ± 0.03^a^	9.07 ± 0.09^b^	8.85 ± 0.09^b^
Tamsulosin	10.32 ± 0.05^c^	8.33 ± 0.08^d^	9.56 ± 0.07^d^

*The functional affinities were determined in the rat tail artery (α_1A_-adrenoceptor subtype), mouse spleen (α_1B_-adrenoceptor subtype), rat aorta (α_1D_-adrenoceptor subtype). Noradrenaline was used as α_1_-adrenergic receptor agonist. The highest pA_2_ values for each receptor subtype are in bold font. Antagonist potency of the tested compounds was expressed as pA_2_ ± S.E.M. pA_2_ was defined as −log of the concentration of an antagonist that doubles the concentration of the agonist necessary to elicit the original submaximal response in the absence of antagonist. pA_2_ values were obtained from the linear regression of Schild plot (Arunlakshana and Schild, 1959). Each value was the mean ± S.E.M. of 4–8 experimental results*.

a *Parés-Hipólito et al. (2006)*.

b *Eltze (1996)*.

c *Jähnichen et al. (2004)*.

d *Eltze et al. (1999)*.

**Table 2B:** **The affinity of tested compounds for α_**1**_-adrenergic receptor subtypes**.

**Compound**	**The affinity for α_1_-adrenergic receptor subtypes**
HBK-14	α_1D_ > α_1A_ > α_1B_
HBK-15	α_1D_ > α_1A_ > α_1B_
HBK-16	α_1D_ > α_1A_ > α_1B_
HBK-17	α_1D_ > α_1A_ > α_1B_
HBK-18	α_1D_ > α_1A_ > α_1B_
HBK-19	α_1A_ > α_1D_ > α_1B_

*The functional affinities were determined in the rat tail artery (α_1A_-adrenoceptor subtype), mouse spleen (α_1B_-adrenoceptor subtype), rat aorta (α_1D_-adrenoceptor subtype)*.

HBK-14, HBK-15, HBK-16, HBK-17, and HBK-18 showed stronger antagonistic properties at α_1D_- than α_1A_- or α_1B_-adrenoceptors. HBK-19 antagonized α_1A_-adrenoceptors stronger than two other subtypes. All compounds antagonized α_1A_-adrenoceptors stronger than α_1B_-subtype. Among the studied compounds the strongest antagonist of α_1A_-adrenoceptor was HBK-19, α_1B_-adrenoceptor—HBK-18, and α_1D_-adrenoceptor—HBK-16.

### The effect on normal electrocardiogram in rats

Table 3 shows the influence of tested compounds on normal ECG in rats.

**Table 3:** **The influence of the tested compounds on ECG**.

**Compound**	**Parameters**	**Time of observation (min)**								
		**0**	**5**	**10**	**15**	**20**	**30**	**40**	**50**	**60**
HBK-14	Beats/min	366.0 ± 10.8	369.8 ± 10.0	367.0 ± 9.9	359.8 ± 9.5	358.4 ± 11.4	359.8 ± 9.6	362.6 ± 11.9	361.6 ± 12.6	364.6 ± 11.2
	PQ (ms)	47.1 ± 1.1	46.0 ± 1.0	46.3 ± 0.9	47.5 ± 1.3	46.3 ± 1.0	48.8 ± 1.9	48.2 ± 2.1	47.3 ± 1.6	48.4 ± 0.9
	QRS (ms)	26.1 ± 0.7	25.8 ± 0.5	27.6 ± 1.0	27.0 ± 0.6	26.3 ± 0.4	26.7 ± 0.9	26.1 ± 0.4	26.7 ± 1.0	27.5 ± 0.7
	QT (ms)	85.8 ± 1.3	85.0 ± 4.1	88.7 ± 1.9	89.0 ± 2.4	88.2 ± 2.9	89.6 ± 1.7	89.0 ± 2.1	87.9 ± 1.2	89.5 ± 2.4
	QT_c_ (ms)	211.7 ± 3.2	209.2 ± 8.3	214.8 ± 5.0	212.9 ± 5.7	212.5 ± 4.6	214.1 ± 5.0	214.1 ± 5.2	210.9 ± 5.1	214.1 ± 4.2
HBK-15	Beats/min	353.8 ± 10.2	365.4 ± 15.3	350.8 ± 10.5	333.9 ± 12.4	327.6 ± 20.3	340.1 ± 17.7	339.5 ± 20.4	350.1 ± 17.9	351.8 ± 15.0
	PQ (ms)	48.7 ± 1.0	48.4 ± 0.5	48.2 ± 1.0	46.6 ± 0.9	47.6 ± 0.5	46.1 ± 0.3	46.1 ± 0.7	47.5 ± 0.7	48.0 ± 1.1
	QRS (ms)	25.7 ± 0.7	26.3 ± 0.9	26.1 ± 1.1	25.6 ± 1.2	26.3 ± 0.5	26.4 ± 0.9	28.0 ± 0.9	26.1 ± 1.2	26.3 ± 0.5
	QT (ms)	87.3 ± 1.4	86.5 ± 1.3	87.2 ± 2.0	87.5 ± 2.0	87.2 ± 0.6	86.7 ± 1.1	87.0 ± 1.5	88.2 ± 0.8	88.5 ± 2.2
	QT_c_ (ms)	212.0 ± 5.0	213.6 ± 7.0	211.3 ± 6.2	207.1 ± 6.8	203.7 ± 7.2	206.4 ± 7.9	206.5 ± 6.6	212.7 ± 6.9	213.9 ± 7.2
HBK-16	Beats/min	329.6 ± 9.8	331.4 ± 12.5	330.1 ± 12.4	319.4 ± 10.3	310.6 ± 9.8	302.8 ± 11.0	294.1 ± 12.2^a^	291.4 ± 13.0^b^	288.0 ± 13.8^b^
	PQ (ms)	64.4 ± 1.1	63.7 ± 1.1	63.6 ± 0.9	63.9 ± 1.1	65.1 ± 1.2	64.4 ± 1.1	65.9 ± 0.8	67.3 ± 1.6	68.2 ± 0.9
	QRS (ms)	21.7 ± 2.2	21.7 ± 2.2	21.7 ± 2.2	21.7 ± 2.2	21.7 ± 2.2	21.7 ± 2.2	21.7 ± 2.2	22.0 ± 2.1	22.3 ± 2.0
	QT (ms)	51.4 ± 3.5	51.2 ± 3.5	51.2 ± 3.3	50.1 ± 3.6	51.4 ± 3.1	52.7 ± 3.7	53.5 ± 2.8	54.7 ± 2.8	55.5 ± 2.4
	QT_c_ (ms)	120.5 ± 8.7	120.7 ± 9.7	120.2 ± 8.8	115.8 ± 9.6	117.3 ± 8.7	119.0 ± 10.3	118.9 ± 8.4	121.1 ± 8.7	122.0 ± 7.7
HBK-17	Beats/min	365.2 ± 15.0	359.4 ± 16.6	348.7 ± 15.0	341.1 ± 13.4	333.5 ± 13.3^b^	327.4 ± 11.8^c^	333.0 ± 10.6^b^	323.8 ± 14.1^c^	320.9 ± 13.4^c^
	PQ (ms)	66.5 ± 3.7	65.0 ± 3.3	64.3 ± 3.4	65.6 ± 3.7	65.9 ± 3.4	67.7 ± 4.0	67.0 ± 3.2	67.4 ± 3.5	67.6 ± 3.3
	QRS (ms)	23.3 ± 1.8	23.3 ± 1.4	23.0 ± 1.7	23.3 ± 2.0	22.7 ± 1.8	22.7 ± 2.0	23.0 ± 1.7	23.3 ± 1.4	23.0 ± 1.7
	QT (ms)	55.2 ± 2.3	55.3 ± 0.9	55.7 ± 1.1	57.4 ± 0.8	57.7 ± 1.8	60.3 ± 1.1	61.3 ± 1.6	60.4 ± 2.0	61.5 ± 2.3
	QT_c_ (ms)	135.8 ± 5.3	135.2 ± 3.3	134.1 ± 3.1	136.5 ± 2.3	135.6 ± 3.5	140.7 ± 3.7	144.3 ± 5.0	140.2 ± 5.4	142.0 ± 5.0
HBK-18	Beats/min	328.0 ± 7.4	306.9 ± 9.0	298.7 ± 8.9	284.7 ± 7.6^a^	273.9 ± 8.0^b^	267.4 ± 17.5^c^	260.3 ± 22.8^c^	258.9 ± 25.4^c^	252.3 ± 25.1^c^
	PQ (ms)	61.3 ± 4.6	61.5 ± 6.3	57.4 ± 4.2	55.8 ± 1.6	51.9 ± 3.8	56.9 ± 3.5	57.4 ± 3.1	59.0 ± 3.8	59.6 ± 4.7
	QRS (ms)	28.1 ± 3.1	30.4 ± 3.2	30.8 ± 3.6	27.0 ± 2.9	29.1 ± 2.7	28.5 ± 2.8	28.6 ± 2.8	27.6 ± 3.0	30.9 ± 3.8
	QT (ms)	73.9 ± 6.2	77.0 ± 7.0	77.7 ± 4.9	76.1 ± 5.8	77.9 ± 4.2	75.3 ± 3.2	76.9 ± 4.9	76.0 ± 2.9	76.7 ± 3.5
	QT_c_ (ms)	172.4 ± 13.5	174.2 ± 16.3	173.4 ± 11.7	166.4 ± 14.5	166.9 ± 11.1	159.0 ± 9.7	160.4 ± 14.4	157.2 ± 9.9	157.1 ± 12.0
HBK-19	Beats/min	355.3 ± 19.7	349.7 ± 25.3	339.1 ± 21.0	280.1 ± 1.1^c^	277.2 ± 5.0^c^	278.2 ± 6.7^c^	332.8 ± 18.9	396.8 ± 31.8	398.0 ± 32.4
	PQ (ms)	56.7 ± 0.7	56.6 ± 0.0	56.0 ± 0.0	58.2 ± 1.8	60.1 ± 1.3	60.1 ± 1.7	59.3 ± 0.7	55.1 ± 0.3	56.3 ± 1.1
	QRS (ms)	35.7 ± 1.0	34.7 ± 1.6	34.0 ± 0.3	32.0 ± 1.2	32.7 ± 0.4	34.3 ± 0.2	32.0 ± 3.1	36.0 ± 1.5	30.3 ± 1.1
	QT (ms)	76.4 ± 8.2	76.9 ± 1.1	77.5 ± 0.5	82.2 ± 2.2	84.3 ± 1.1	82.2 ± 0.4	81.5 ± 2.5	76.5 ± 0.5	80.2 ± 0.4
	QT_c_ (ms)	186.9 ± 24.6	185.5 ± 9.5	184.2 ± 6.9	177.6 ± 4.4	181.1 ± 4.1	177.0 ± 3.0	192.2 ± 11.2	196.2 ± 6.9	206.3 ± 9.6

*Rats were anesthetized intraperitoneally (i.p.) with thiopental (75 mg/kg). The compounds were administered (i.p.) at the ED_*84*_ obtained in prophylactic adrenaline-induced arrhythmia i.e., 6.154 mg/kg (HBK-14), 20.218 mg/kg (HBK-15), 0.363 mg/kg (HBK-16), 0.504 mg/kg (HBK-17), 0.325 mg/kg (HBK-18), 0.444 mg/kg (HBK-19). The data are expressed as mean ± S.E.M. Statistical analysis: one-way ANOVA with repeated measurements, Dunnet post-hoc test*,

a *p < 0.05*,

b *p < 0.01*,

c *p < 0.001 vs. initial values. n = 4–6 rats per group*.

HBK-14 and HBK-15 administered at the dose 6.154 and 20.218 mg/kg, respectively did not influence the ECG parameters throughout the experiment. HBK-16 at the dose 0.363 mg/kg did not influence PQ interval, QRS complex or QTc interval but it significantly reduced heart rate by 11−13%, since the 40th min of the observation. Similarly, HBK-17 at the dose 0.504 mg/kg significantly decreased heart rate by 9−12%, 20 min after administration, without affecting PQ interval, QRS complex or QTc interval. HBK-18 at the dose 0.325 mg/kg significantly reduced the heart rate by 13−23%, since the 15th min of the observation and did not affect PQ interval, QRS complex or QTc interval. HBK-19 at the dose 0.444 mg/kg between the 15th and 30th min after administration reduced heart rate by 21−22% without the influence on PQ interval, QRS complex, or QTc interval.

### Prophylactic antiarrhythmic activity in adrenaline-, barium chloride-, and calcium chloride-induced arrhythmias

**Figure 2:**
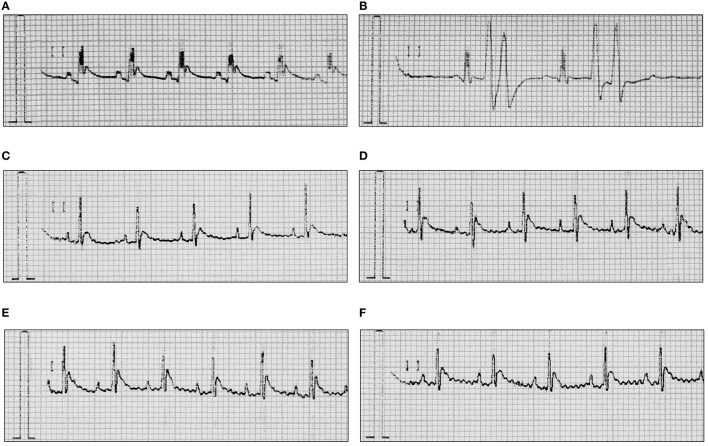
**Representative ECGs after treatment with adrenaline and/or HBK-16, HBK-17, HBK-18, and HBK-19 in rats**. **(A)** Normal reading (Control). **(B)** Arrhythmia control—adrenaline (20 μg/kg, i.v.). **(C)** Adrenaline-induced arrhythmia (20 μg/kg, i.v.)+HBK-16 (0.3 mg/kg, i.v. injection 45 min prior to adrenaline). **(D)** Adrenaline-induced arrhythmia (20 μg/kg, i.v.)+HBK-17 (0.3 mg/kg, i.v. injection 45 min prior to adrenaline). **(E)** Adrenaline-induced arrhythmia (20 μg/kg, i.v.)+HBK-18 (0.3 mg/kg, i.v. injection 45 min prior to adrenaline). **(F)** Adrenaline-induced arrhythmia (20 μg/kg, i.v.)+HBK-19 (0.3 mg/kg, i.v. injection 45 min prior to adrenaline).

**Figure 3:**
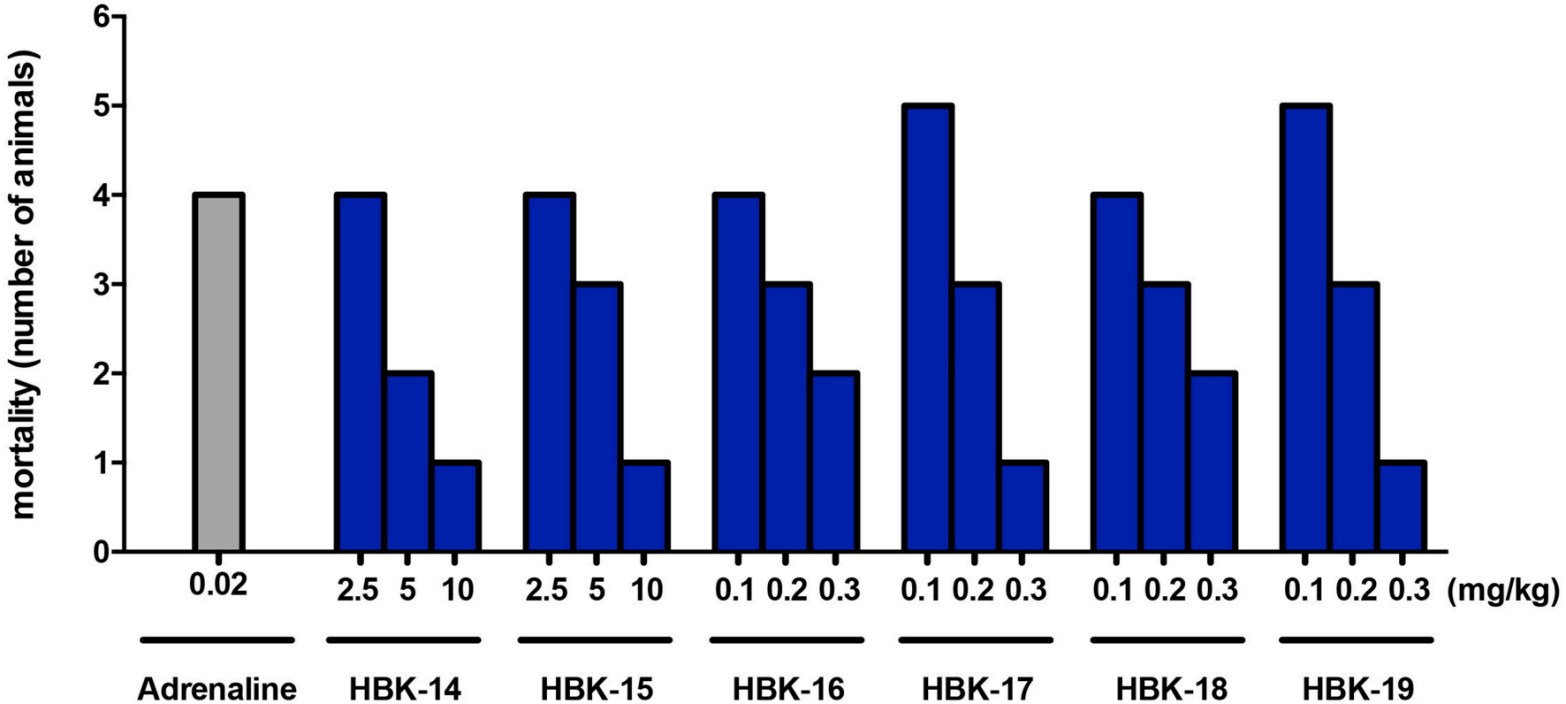
**The effect of adrenaline and studied compounds on mortality of rats in prophylactic adrenaline-induced arrhythmia**. Rats were anesthetized with intraperitoneal (i.p.) injection of thiopental (75 mg/kg). The tested compounds were administered (i.p.) 45 min before the experiment. The observation was carried out for 15 min after the intravenous injection of adrenaline (20 μg/kg). *n* = 5−6 animals per group.

In anesthetized rats i.v. injection of adrenaline (20 μg/kg) caused atrioventricular disturbances, ventricular and supraventricular extrasystoles in 100% of the animals, which led to the death of ~70% of the animals. The studied compounds administered 45 min (i.p.) prior to adrenaline, decreased the number of extrasystoles and mortality (Figures 2, 3, Table 4). Table 5 shows the ED_50_ values (doses producing 50% inhibition of premature ventricular contractions).

**Table 4:** **The antiarrhythmic activity of 2-methoxy phenylpiperazine derivatives in prophylactic model of adrenaline induced arrhythmia**.

**Compound**	**Dose (mg/kg)**	**Extrasystoles (%)**	**Bigeminy (%)**	**Bradycardia (%)**	**Blocks (%)**
Adrenaline	–	66.7	33.3	66.7	50.0
HBK-14	2.5	100.0	50.0	83.3	83.3
	5	50.0	33.3	50.0	33.3
	10	16.7	16.7	50.0	33.3
HBK-15	2.5	66.7	33.3	66.7	66.7
	5	50.0	16.7	66.7	50.0
	10	33.3	16.7	50.0	33.3
HBK-16	0.1	83.3	33.3	66.7	50.0
	0.2	33.3	16.7	50.0	33.3
	0.3	16.7	–	33.3	16.7
HBK-17	0.1	66.7	50.0	67.7	67.7
	0.2	50.0	33.3	50.0	33.3
	0.3	16.7	16.7	33.3	16.7
HBK-18	0.1	83.3	50.0	66.7	66.7
	0.2	50.0	16.7	50.0	33.0
	0.3	16.7	12.0	16.3	16.3
HBK-19	0.1	66.7	33.3	66.7	50.0
	0.2	50.0	16.7	33.3	33.3
	0.3	16.7	–	16.7	16.7

*Rats were anesthetized with intraperitoneal (i.p.) injection of thiopental (75 mg/kg). The tested compounds were administered (i.p.) 45 min before the experiment. The observation was carried out for 15 min after the intravenous injection of adrenaline (20 μg/kg) i.e., during the first 2 min, in the 5, 10, and 15th min. n = 5−6 animals per group*.

**Table 5:** **Prophylactic antiarrhythmic activities of tested compounds and carvedilol in adrenaline-induced arrhythmia**.

**Compound**	**ED_50_(mg/kg)**
HBK-14	3.88 (2.68−5.61)
HBK-15	4.80 (2.32−9.93)
HBK-16	0.20 (0.10−0.39)
HBK-17	0.20 (0.12−0.35)
HBK-18	0.18 (0.11−0.29)
HBK-19	0.21 (0.13−0.34)
Carvedilol	0.36 (0.16−0.80)

*Rats were anesthetized with intraperitoneal (i.p.) injection of thiopental (75 mg/kg). The tested compounds were administered (i.p.) 45 min before the experiment. The observation was carried out for 15 min after the intravenous injection of adrenaline (20 μg/kg) i.e., during the first 2 min, in the 5, 10, and 15th min. The ED_50_ values with confidence limits were calculated according to the methods described by Litchfield and Wilcoxon (1949). Each value was obtained from three experimental groups. n = 5−6 animals per group*.

The injection (i.v.) of barium chloride (32 mg/kg) caused rapid ventricular extrasystoles in all animals (100%), which led to the death within 3−5 min. Lower dose of barium chloride (10 mg/kg) caused ventricular extrasystoles in around 60% of rats and did not lead to the death of animals. We did not observe a reproducible negative effect on heart rhythm when barium chloride was used at the dose 10 mg/kg. None of the tested compounds administered (i.p.) 45 min before barium chloride were active in barium chloride-induced model of arrhythmia (data not shown).

In anesthetized rats injection (i.v.) of calcium chloride (140 mg/kg) caused rapid ventricular fibrillation in all animals (100%), which led to death within 3−5 min. The intravenous administration of calcium chloride (25 mg/kg) caused ventricular fibrillation in all animals, but did not lead to the death of rats. None of the tested compounds administered (i.p.) 45 min prior to calcium chloride were active in the above model of arrhythmia (data not shown).

### Therapeutic antiarrhythmic activity in adrenaline-induced arrhythmia

**Figure 4:**
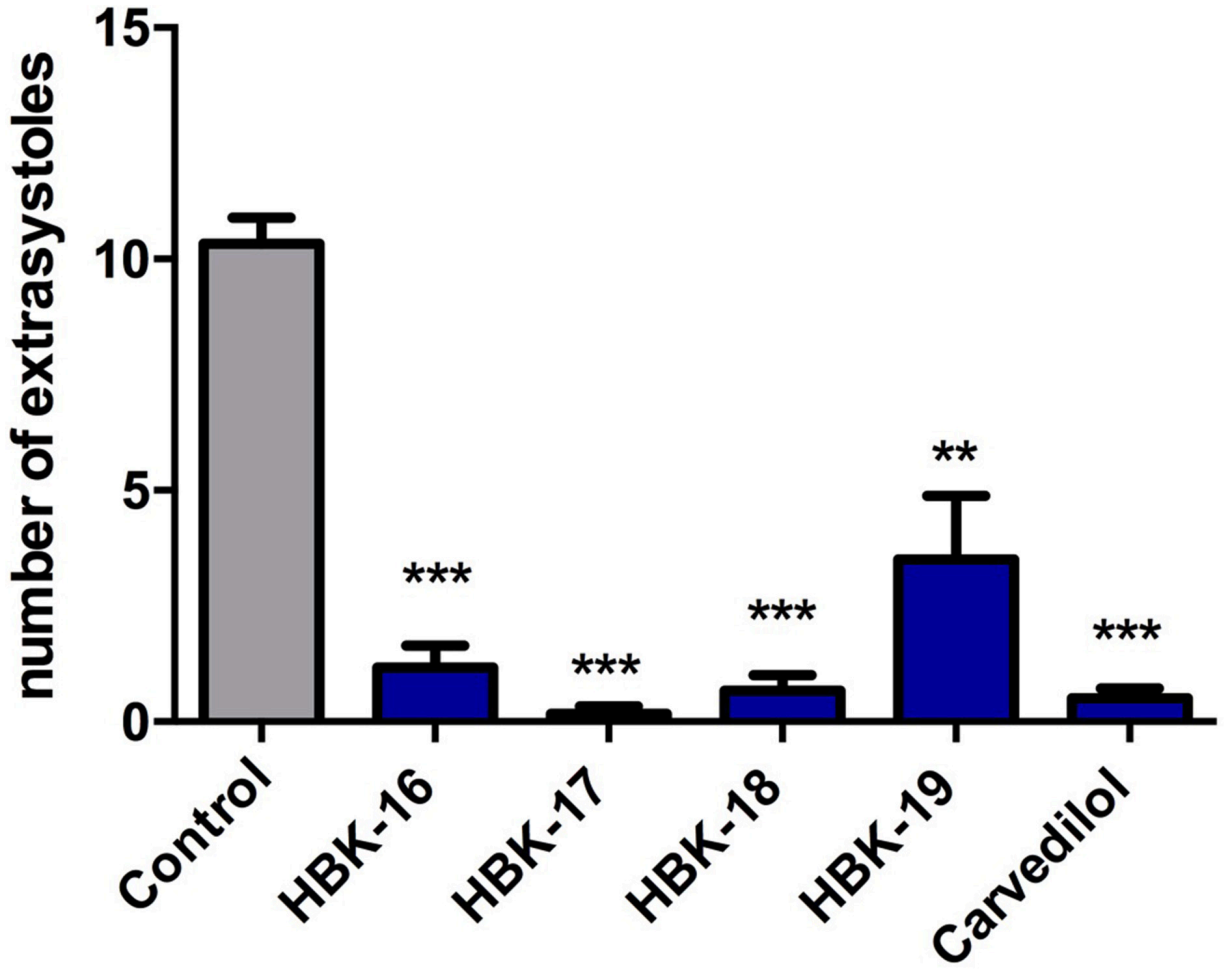
**Therapeutic antiarrhythmic activities of tested compounds in adrenaline-induced arrhythmia**. Rats were anesthetized with intraperitoneal injection of thiopental (75 mg/kg). The tested compounds were administered intravenously (i.v.) at the ED_84_ obtained in prophylactic adrenaline-induced arrhythmia i.e., 0.363 mg/kg (HBK-16), 0.504 mg/kg (HBK-17), 0.325 mg/kg (HBK-18), 0.444 mg/kg (HBK-19), 0.979 mg/kg (carvedilol). Data are reported as means ± S.E.M. Statistical analysis: Student's *t*-test; ^**^p < 0.01, ^***^p < 0.001. *n* = 5−6 animals per group.

All tested compounds administered i.v. at the peak of adrenaline-induced arrhythmia (20 μg/kg) reduced the number of premature ventricular contractions (Figure 4).

### Influence on blood pressure in normotensive rats

Table 6 presents the lowest dose of each tested compound, which significantly lowered systolic and diastolic blood pressure in rats.

**Table 6:** **The hypotensive activity of 2-methoxyphenylpiperazine derivatives in normotensive rats**.

**Compound**	**Dose (mg/kg)**	**Pressure**	**Time of observation (min)**							
			**Control**	**5**	**10**	**20**	**30**	**40**	**50**	**60**
HBK-14	0.625	Systolic	138.3 ± 6.8	135.2 ± 7.3	131.2 ± 6.8^c^	127.3 ± 6.6^c^	125.3 ± 7.0^c^	124.2 ± 7.0^c^	127.3 ± 6.3^c^	128.2 ± 5.4^c^
		Diastolic	106.7 ± 4.3	105.2 ± 6.1	103.0 ± 5.8	97.3 ± 6.0^c^	95.2 ± 6.1^c^	92.3 ± 6.2^c^	94.0 ± 5.8^c^	98.0 ± 5.1^c^
HBK-15	5.0	Systolic	139.0 ± 2.7	121.2 ± 4.8^c^	116.3 ± 6.0^c^	112.0 ± 5.5^c^	112.2 ± 4.2^c^	112.5 ± 4.4^c^	113.2 ± 3.7^c^	117.3 ± 3.0^c^
		Diastolic	110.3 ± 2.2	96.3 ± 3.7^c^	92.0 ± 4.3^c^	88.5 ± 3.5^c^	88.3 ± 2.9^c^	91.0 ± 3.2^c^	90.2 ± 2.7^c^	93.0 ± 1.2^c^
HBK-16	0.1	Systolic	124.7 ± 3.1	123.2 ± 3.3	121.2 ± 3.2	114.8 ± 2.0^b^	113.0 ± 2.4^c^	110.3 ± 2.1^c^	110.8 ± 2.0^b^	110.7 ± 1.9^c^
		Diastolic	97.0 ± 4.3	95.0 ± 5.1	93.7 ± 5.1	91.3 ± 5.0^b^	89.8 ± 5.2^a^	87.7 ± 4.7^b^	88.0 ± 4.3^c^	87.7 ± 4.6^b^
HBK-17	0.1	Systolic	136.8 ± 3.7	132.7 ± 4.8	130.5 ± 4.7	125.5 ± 4.9^b^	124.2 ± 4.4^b^	121.7 ± 3.9^b^	121.5 ± 3.4^a^	121.2 ± 3.9^b^
		Diastolic	110.7 ± 4.6	108.2 ± 5.2	105.8 ± 4.3	99.3 ± 4.7^a^	96.8 ± 4.0^b^	94.0 ± 3.7^b^	93.3 ± 4.0^b^	94.5 ± 4.3^b^
HBK-18	0.01	Systolic	125.7 ± 8.2	117.7 ± 9.1	112.7 ± 7.7	96.5 ± 8.9^b^	93.8 ± 8.7^c^	91.8 ± 10.0^b^	90.7 ± 8.6^b^	90.2 ± 9.2^a^
		Diastolic	94.3 ± 6.4	90.7 ± 7.1	85.5 ± 7.5	79.5 ± 7.8^a^	78.3 ± 7.2^a^	78.8 ± 7.0^b^	78.7 ± 5.6^c^	77.5 ± 6.0^b^
HBK-19	0.625	Systolic	127.0 ± 4.0	121.0 ± 4.0^b^	116.0 ± 3.5^c^	111.5 ± 3.2^c^	110.0 ± 2.3^c^	107.5 ± 3.2^c^	106.0 ± 3.5^c^	106.0 ± 4.0^c^
		Diastolic	98.0 ± 4.6	94.5 ± 2.6	87.5 ± 0.4^c^	89.0 ± 2.3^b^	87.0 ± 2.9^c^	84.5 ± 1.4^c^	83.5 ± 1.4^c^	84.0 ± 0.6^c^

*Rats were anesthetized intraperitoneally (i.p.) with thiopental (75 mg/kg). The compounds were administered (i.p.). The results present the lowest hypotensive dose. The data are the means of six experiments ± S.E.M. Statistical analysis: one-way ANOVA test with repeated measurements, Dunnet post-hoc test*.

a *p < 0.05*,

b *p < 0.01*,

c *p < 0.001 vs. control values. n = 6 rats per group*.

HBK-14 at the dose 0.625 mg/kg, 10 min after injection, significantly reduced systolic blood pressure by 5−10%, and diastolic blood pressure by 9−14% since the 20th min of the observation. HBK-15 at the dose 5.0 mg/kg significantly reduced systolic blood pressure by 13−19%, and diastolic blood pressure by 13−20%, 5 min after administration. HBK-16 at the dose 0.1 mg/kg, 20 min after administration, significantly reduced systolic blood pressure by 8−11% and diastolic blood pressure by 6−10%. HBK-17 at the dose 0.1 mg/kg significantly reduced systolic blood pressure by 8−11% and diastolic blood pressure by 10−16%, 20 min after administration. HBK-18 at the dose 0.01 mg/kg, from the 20th min of the observation, significantly reduced systolic and diastolic blood pressure by 23−28 and 16−18%, respectively. HBK-19 at the dose 0.625 mg/kg since the 5th min after i.p. administration, significantly reduced systolic blood pressure by 5−17%, whereas diastolic blood pressure by 9−15% since the 10th min of the observation.

### Influence on blood vasopressor response in rats

**Figure 5:**
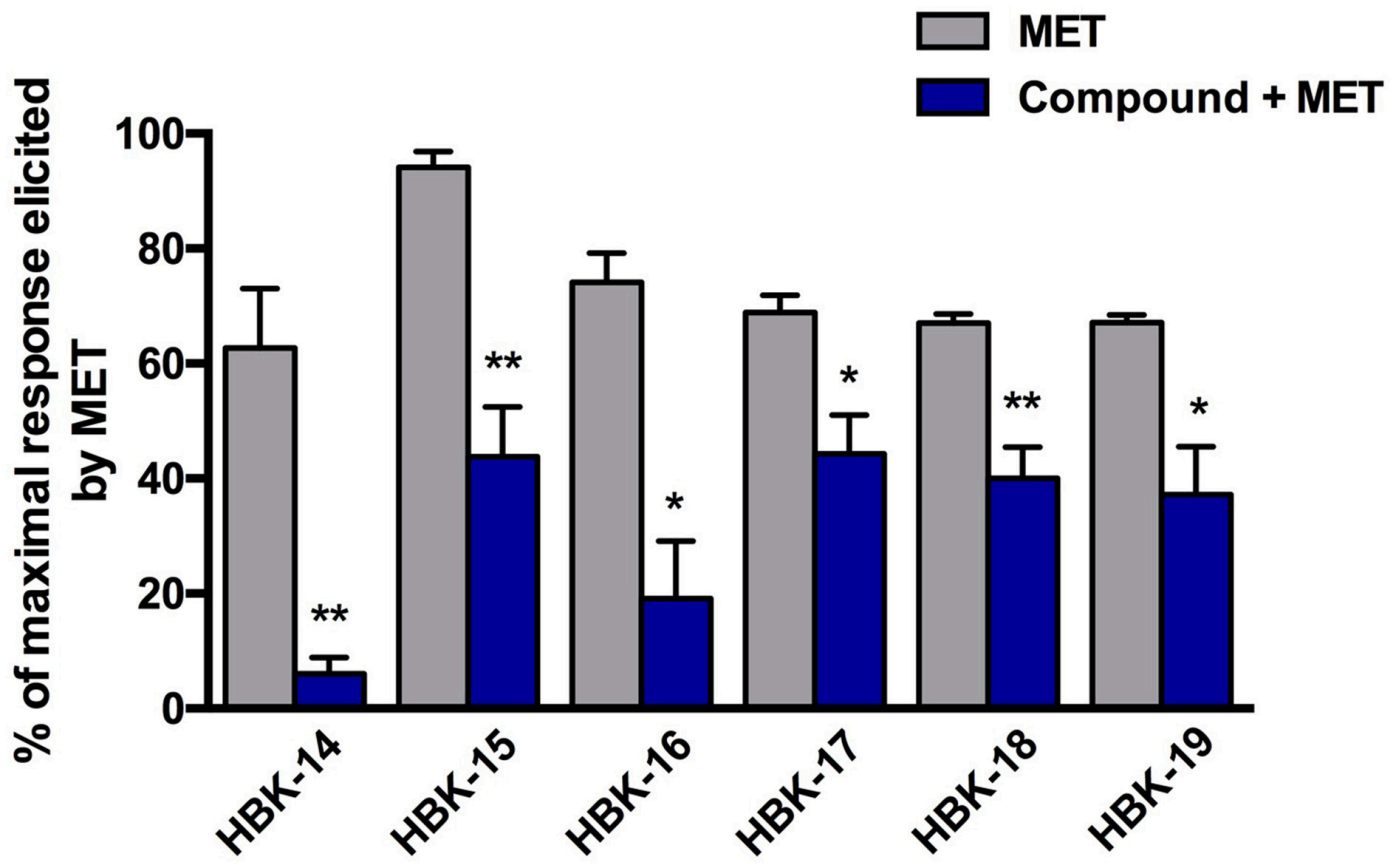
**The effect of tested compounds on the blood pressure response to methoxamine**. Rats were anesthetized with intraperitoneal injection of thiopental (75 mg/kg). The studied compounds were administered intravenously (i.v.), at the lowest hypotensive doses i.e., 0.625 mg/kg (HBK-14), 5.0 mg/kg (HBK-15), 0.1 mg/kg (HBK-16), 0.1 mg/kg (HBK-17), 0.01 mg/kg (HBK-18), 0.625 mg/kg (HBK-19). Methoxamine (MET) was administered at the dose 150 μg/kg (i.v.). All values represent means ± S.E.M. Statistical analysis: Student's *t*-test; ^*^p < 0.05, ^**^p < 0.01, compared to the initial maximal response (obtained before the administration of tested compounds). *n* = 5−6 animals per group.

In the control group the increase of blood pressure after methoxamine (150 μg/kg) was ranging from 62.7 ± 10.4 to 94.2 ± 2.7 mmHg. Figure 5 shows that all tested compounds at the lowest hypotensive doses, significantly antagonized the pressor response to methoxamine.

### Influence on lipid peroxidation in rat brain homogenate

Carvedilol, HBK-16, HBK-17, HBK-18, and HBK-19 inhibited lipid peroxidation (Table 7). HBK-14 and HBK-15 were inactive in this test.

**Table 7:** **The influence of the tested compounds on lipid peroxidation in rat brain homogenate—antioxidant effect**.

**Compound**	**Absorbance**	**% reduction of absorbance (% antioxidant activity)**
HBK-14	0.939 ± 0.007	−4.10
HBK-15	0.891 ± 0.008	1.22
HBK-16	0.752 ± 0.007	16.63
HBK-17	0.773 ± 0.009	14.30
HBK-18	0.781 ± 0.004	13.41
HBK-19	0.698 ± 0.007	22.62
Carvedilol	0.094 ± 0.002	89.58

*The rates of membrane lipid peroxidation were measured using rat brain homogenate by the formation of thiobarbituric acid reactive substances (TBARs). The studied compounds were tested at a concentration of 10^−3^ M. The TBARs were measured at 532 nm*.

## Discussion

We found that the studied 2-methoxyphenylpiperazine derivatives that possessed stronger α_1A_-adrenolytic properties (i.e., HBK-16, HBK-17, HBK-18, and HBK-19) were the most active compounds in adrenaline-induced arrhythmia. Their antiarrhythmic (but not antioxidant) activity was comparable to that of carvedilol. The tested compounds showed hypotensive effect resulting from their α_1_-adrenolytic properties. HBK-19 was the only compound in the group that did not lower blood pressure at antiarrhythmic doses.

Scientists reported that 2-methoxyphenylpiperazine derivatives often interact with adrenergic receptors (Handzlik et al., 2008; Kubacka et al., 2013a; Rapacz et al., 2014, 2015a,b). Thus, we evaluated the affinity of the studied compounds for α_1_-and β_1_-adrenoceptors. The radioligand studies revealed that HBK-16, HBK-17, HBK-18, and HBK-19 possessed high affinity for α_1_-, but not β_1_-adrenoceptors. This is in agreement with our previous experiments on 2-methoxyphenylpiperazine derivatives i.e., HBK-14 and HBK-15, which showed no affinity for β_1_- and high affinity for α_1_-adrenoreceptors (Pytka et al., 2015).

Studies proved that cardiac myocytes functionally express α_1A_- and α_1B_-adrenoceptors. O'Connell et al. (2003) demonstrated that despite the presence of α_1D_-adrenoceptors mRNA, rodent cardiac myocytes did not express α_1D_-subtype protein by binding. However, Chalothorn et al. (2003) showed that α_1D_-adrenoceptors might be expressed in the coronary vasculature. Thus, α_1A_- and α_1B_-adrenoceptors may play important role in the development of arrhythmias induced by catecholamines, while α_1D_-subtype in ischemia-induced arrhythmias.

Scientists indicated that agents which block α_1A_-adrenoceptors may have antiarrhythmic potential (Hieble, 2000). Harrison et al. (1998) showed that hearts from transgenic rats expressing constitutively active α_1B_-adrenoceptors, and having 50% reduced α_1A_-mRNA levels, were less sensitive to ischemia-induced ventricular tachycardia than normal rats. Lee and Rosen (1993) proved that the blockade of α_1B_ receptors by chlorethylclonidine increased the amplitude of delayed afterdepolarizations induced by calcium and phenylephrine. Altogether, these findings suggest that agents, which block α_1A_-adrenoceptors stronger than α_1B_ subtype, may have antiarrhythmic potential.

Therefore, we determined the selectivity of studied compounds at α_1_-adrenoceptor subtypes. Biofunctional assays revealed that all compounds competitively blocked α_1A_, α_1B_, and α_1D_ subtypes. HBK-19 and HBK-16 were the strongest α_1A_-adrenoceptor antagonists, while HBK-14 and HBK-15 were the weakest. Although we did not find highly selective compounds, all 2-methoxyphenylpiperazine derivatives antagonized α_1A_-adrenoceptors stronger than α_1B_ subtype. HBK-19 showed the greatest difference in pA_2_ values—it blocked α_1A_-adrenoceptors around seven-fold stronger than α_1B_ subtype.

Since all studied compounds blocked α_1A_- and α_1B_-adrenoceptors, we decided to examine their antiarrhythmic activity. We also investigated whether antiarrhythmic activity depended on the strength of α_1A_-adrenoceptor blockade or the differences between pA_2_ values for α_1A_- and α_1B_-adrenoceptors. To determine antiarrhythmic effect, we used three models of arrhythmia i.e., adrenaline-, barium chloride-, and calcium chloride-induced.

All compounds showed antiarrhythmic activity in adrenaline-induced model of arrhythmia, and reduced mortality of rats. HBK-18 possessed the strongest prophylactic antiarrhythmic properties, but ED_50_ values for HBK-16, HBK-17, and HBK-19 were also very low. Except for HBK-14 and HBK-15, prophylactic antiarrhythmic activities of compounds in adrenaline-induced arrhythmia were comparable to that of carvedilol (reference drug). We think that the weak antiarrhythmic activity of HBK 14 and HBK 15 may be due to their weaker α_1_-adrenolytic properties (see Table 2), which are crucial for antiarrhythmic effect in the applied model of arrhythmia. Since the compounds used in the experiments did not present potent selectivity toward different subtypes of α_1−_adrenoceptors, we cannot definitely conclude which receptor subtype should be primarily blocked to achieve antiarrhythmic effect. Although, HBK-19 showed the greatest difference in pA_2_ values for the α_1A_- and α_1B_ receptor subtypes, it did not possess the strongest antiarrhythmic properties. Similarly, according to the studies performed by Koshimizu et al. (2004), the pA_2_ value for α_1A−_adrenoceptor subtype for carvedilol was 9.0, whereas for α_1B−_adrenoceptor was 10.0. Carvedilol showed comparable properties in adrenaline-induced arrhythmia model. Thus, we claim that the potent blockade of α_1A_-receptor subtype is essential to attenuate adrenaline-induced arrhythmia, but the role of α_1B_-adrenoceptor blockade needs further studies. Carvedilol is a potent β_1_- and α_1_-adrenoceptors blocker with antioxidant activity. Surprisingly, despite the fact that carvedilol blocked both β_1_- and α_1_-adrenoceptors, and the studied compounds only α_1_-adrenoceptors, their antiarrhythmic effect was comparable. Therefore, we suggest that this may indicate more important role of α_1_- than β_1_-adrenoceptors blockade in adrenaline-induced arrhythmia model.

Arrhythmia models induced by calcium or sodium chloride are associated with the changes in intracellular ion concentration. These changes are ion channel dependent, and their dynamics and amplitude are high. In arrhythmias induced by adrenaline, the stimulation of adrenergic receptors also leads to ion level changes (primarily Ca^2+^), but these changes are not as rapid. Their amplitude and dynamics are much lower than the above. None of the compounds showed activity in barium chloride- or calcium chloride-induced arrhythmias. Therefore, we can assume that the blockade of sodium or calcium channels was not the predominant mechanism of antiarrhythmic effect of the studied compounds.

We decided to test therapeutic antiarrhythmic potential of the most active compounds (i.e., HBK-16, HBK-17, HBK-18, and HBK-19) in adrenaline-induced model of arrhythmia. The studied compounds restored normal heart rhythm administered at the peak of arrhythmia, but the strongest therapeutic antiarrhythmic activity showed HBK-18. The results of this experiments correlate with the results obtained in prophylactic adrenaline-induced arrhythmia model.

Antiarrhythmic agents have proarrhythmic potential, thus we evaluated the influence of studied compounds on normal ECG in rats. Only HBK-14 and HBK-15 did not influence ECG at ED_84_ obtained in prophylactic adrenaline-induced arrhythmia model. The rest of compounds significantly decreased heart rate. Williamson et al. (1994) proved that stimulation of α_1A_-adrenoceptors resulted in positive chronotropic effect. This suggests that the decrease in heart rate observed after treatment with HBK-16, HBK-17, HBK-18, and HBK-19 was a result of α_1A_ receptor blockade.

The QT interval represents electrical depolarization and repolarization of ventricles. The prolongation of QT interval indicates the potential of a drug to cause ventricular tachyarrhythmias like *torsades de pointes*. The QTc adjusts the QT interval for heart rate extremes. In this study we used Bazett's equation to calculate QTc. Nevertheless, we need to point out that even though Bazett's formula is very often used for QT correction, it has many limitations (e.g., over- and under-correction of high or low heart rhythms). Rodents' heart rate values can be several times higher than those observed in humans (Kmecova and Klimas, 2010). Since heart rhythm significantly influences QTc, this might be the reason for the observed differences in baseline QTc in our experiments. We showed that none of the compounds affected QTc at ED_84_, therefore we can assume that they did not have proarrhythmic potential at antiarrhythmic doses.

Our findings are in agreement with the results obtained by other researchers, showing that phenylpiperazine derivatives possessed α_1_-adrenolytic properties, as well as prophylactic and/or therapeutic activity in adrenaline-induced arrhythmia (Dylag et al., 2004; Handzlik et al., 2012; Kubacka et al., 2013a,b).

When discussing adrenaline-induced arrhythmias we could neglect the role of α_1D_-adrenoceptors, since their blockade should not have a direct influence on cardiac myocytes. Nevertheless, in animal studies on drug candidates, we cannot entirely ignore the role of α_1D_-subtype. α_1D_-Adrenoceptors blockade in blood vessels might significantly lower blood pressure, which due to the baroreflex might increase heart rate, and contribute to arrhythmia.

Since all studied compounds blocked α_1D_-adrenoceptor, and these receptors among others regulate blood pressure, we evaluated their influence on blood pressure in rats. All tested compounds showed hypotensive properties. HBK-18 showed the strongest hypotensive activity, while HBK-15 the weakest. Interestingly, the results of this experiment did not correlate with the functional bioassays, where the strongest α_1D_-adrenoceptor blocking properties showed HBK-16. We suspect that this may be due to the differences in receptor binding dynamics, but this issue would require further experiments. Regarding the case of HBK-19, the lowest dose that reduced blood pressure was around three-fold higher than ED_50_ value in prophylactic adrenaline-induced arrhythmia. For antiarrhythmic drugs, hypotensive activity is not desirable, since α_1_-adrenoceptor blockers acting in the periphery, may induce reflex tachycardia, and contribute to cardiac arrhythmias. In our opinion, the lack of hypotensive properties at antiarrhythmic doses makes HBK-19 the most interesting compound in the studied group. Our results suggest that the receptor profile of HBK 19 (the highest affinity for α_1A_ and the lowest for α_1D_) is the most beneficial in preventing adrenaline-induced arrhythmia. Interestingly, this suggests that in *in vivo* conditions the selectivity between α_1A_ and α_1D_ is much more important in achieving the optimal profile of α_1_-adrenolytics acting as antiarrhythmic agents, than the selectivity between α_1A_ and α_1B_.

In order to prove that hypotensive properties of tested compounds were a result of their α_1_-adrenolytic properties, we performed the experiments with methoxamine (selective α_1_-adrenoceptor agonist). Drugs that selectively block α_1_-adrenergic receptors significantly inhibit pressor response to methoxamine. All studied compounds blocked the effect caused by methoxamine, thus we concluded that their hypotensive activity was due to α_1_-adrenolytic properties.

Oxidative stress plays an important role in arrhythmias (Dudek et al., 2014; Sovari, 2016). Reactive oxygen species (ROS) prolong action potential duration, induce early afterdepolarizations, and delayed afterdepolarizations in rats and guinea-pigs (Beresewicz and Horackova, 1991). Scientists indicated that oxidative stress activated Ca2+/CaM-dependent kinase II (CaMKII), and consequently caused arrhythmias (Xie et al., 2009). Rabbits with cardiac hypertrophy pretreated with CaMKII inhibitor were less likely to develop ventricular arrhythmias (Ke et al., 2007). Kirshenbaum et al. (1990) showed that vitamin E (antioxidant) protected rats with chronic heart hypertrophy against adrenaline-induced arrhythmias. This suggests that oxidative stress plays role in adrenaline-induced arrhythmias.

Given the significant antiarrhythmic effect of the studied compounds, we decided to investigate whether 2-methoxyphenylpiperazine derivatives possess additional antioxidant activity. Strong antioxidant activity might have contributed to their antiarrhythmic effect. This would explain their significant effect in adrenaline-induced model of arrhythmia. Our experiments showed that among all studied compounds only HBK-16, HBK-17, HBK-18, and HBK-19 weakly inhibited lipid peroxidation. The effect elicited by HBK-19 was the strongest in the group. However, its activity was around eight-fold weaker than the effect caused by carvedilol. Although HBK-16, HBK-17, HBK-18, and HBK-19 possessed weaker antioxidant properties than carvedilol, they showed stronger antiarrhythmic activity. This confirms that in adrenaline-induced arrhythmia model, the blockade of α_1_-adrenoceptors is more important for antiarrhythmic activity than antioxidant properties of the compound. Moreover, our findings suggest that antiarrhythmic properties of studied compounds resulted predominantly from α_1_-adrenolytic properties.

The levels of cardiac α_1_-adrenoceptor are around 10-fold higher in rats than in humans. This may suggest that the role of α_1_-adrenoceptor blockade in arrhythmia is not as significant in humans. Interestingly, despite lower expression of α_1_-adrenoceptors in human heart, scientists proved that they play a significant role in arrhythmias (Furushima et al., 2001). Kurtzwald-Josefson et al. (2014) identified a contribution of α-adrenergic pathway to pathogenesis of catecholamine-induced arrhythmia, and suggested α-blockade as an effective therapy in the murine model of catecholaminergic polymorphic ventricular tachycardia. The Authors suggested α-adrenolytics as an alternative treatment in humans resistant to β-blockers. Thus, it would be reasonable to keep searching for antiarrhythmic agents among α_1_-adrenolytics.

Since structural similarity of studied compounds reduces the likelihood of various mechanisms of action, in future studies we plan to investigate another set of structurally similar 2-methoxyphenylpiperazine derivatives with higher selectivity toward α_1A_-adrenoceptor subtype. This might give more insight into the role of α_1A_-adrenoceptor subtype in antiarrhythmic effect.

In conclusion, the studied 2-methoxyphenylpiperazine derivatives possessed high affinity for α_1_-adrenoceptors and competitively antagonized α_1A_, α_1B_, and α_1*D*_ receptor subtypes. The compounds that possessed stronger α_1A_-adrenolytic properties (i.e., HBK-16, HBK-17, HBK-18, and HBK-19) were the most active compounds in adrenaline-induced arrhythmia. We suggest that their antiarrhythmic activity results predominantly from strong α_1A_-adrenolytic properties.

## Author contributions

Conceived and designed the experiments: KP, SM, JS, BF. Performed the experiments: KP, SM, KL, EŻ, MK, AS, AD, JŚniecikowska, MZ. Analyzed the data: KP, SM, KL, EŻ, AO, AG, JŚmieja. Contributed reagents/materials/analysis tools: AW, HM. Wrote the paper: KP, SM, AO, KL, EŻ, MK.

## Funding

This study was supported by Jagiellonian University grants number K/DSC/000040 and K/DSC/001955. This work was partially supported by NCN grant DEC- 2013/11/B/ST7/01713 and Silesian University BK grant 227/RAu1/2015/1.

### Conflict of interest statement

The authors declare that the research was conducted in the absence of any commercial or financial relationships that could be construed as a potential conflict of interest.
